
JOURNAL INFORMATION
==============================
NLM Title Abbreviation: Eur Psychiatry
Journal ID: Eur Psychiatry
NLM Journal ID: 9111820
Journal ID: EPA

European Psychiatry

ISSN: 0924-9338
EISSN: 1778-3585
Publisher: Cambridge University Press

ARTICLE INFORMATION
==============================
PMCID: PMC9412713
DOI: 10.1192/j.eurpsy.2020.8
Article ID: S0924933820000085
Article version: 1
Subjects: Abstracts

Joint Symposium

Publication date: 2020 Jul
Electronic publication date: 2020 Sep 4
Volume: 63
Issue: Suppl 1
Issue title: Abstracts of the 28th European Congress of Psychiatry
First page: S594
Last page: S595
Copyright: © The Author(s) 2020
Copyright year: 2020
Copyright holder: The Author(s)
License: This is an Open Access article, distributed under the terms of the Creative Commons Attribution licence (http://creativecommons.org/licenses/by/4.0/), which permits unrestricted re-use, distribution, and reproduction in any medium, provided the original work is properly cited.
License URL: https://creativecommons.org/licenses/by/4.0/

Page count: 2

==============================

Article ID: JS006
Subjects: ECNP symposium hosted by the EPA: transdiagnostic approaches in Neuropsychiatry

Genomic insights into the overlap between psychiatric disorders

Owen M.
Cardiff University , Mrc Centre For Neuropsychaitric Genetics and Genomics., Cardiff , United Kingdom

Article ID: JS008

The prism project: a quantitative biological approach across neuropsychiatric disorders

Kas M.
University of Groningen , Groningen Institute for Evolutionary Life Sciences, Groningen , Netherlands
Keywords: schizophrenia, Alzheimer Disease, Precision Medicine, Quantitative biology

Conflict of interest:

No

Article ID: JS010
Subjects: EPA-EUFAS symposium - will the opioid crisis hit Europe and what to do?

Co-morbidity: opioid use disorders, pain, depression and suicide

Melich M. Torrens
Universitat Autonoma de Barcelona, Psychiatry , Barcelona , Spain
Keywords: opioid, Dépression, pain, Suicide

Conflict of interest:

No

